# Toward the formation of a Companion Animal Parasite Council for the Tropics (CAPCT)

**DOI:** 10.1186/s13071-015-0884-4

**Published:** 2015-05-13

**Authors:** Rebecca J. Traub, Peter Irwin, Filipe Dantas-Torres, Gabriela Pérez Tort, Norma Vollmer Labarthe, Tawin Inpankaew, Mukulesh Gatne, Bui Khanh Linh, Volker Schwan, Malaika Watanabe, Susanne Siebert, Norbert Mencke, Roland Schaper

**Affiliations:** The Faculty of Veterinary and Agricultural Sciences, The University of Melbourne, Melbourne, Parkville, Victoria 3052 Australia; College of Veterinary Medicine, School of Veterinary and Life Sciences, Murdoch University, Murdoch, Western Australia 6150 Australia; Department of Immunology, Centro de Pesquisas Aggeu Magalhães, Fundação Oswaldo Cruz, 50740465 Recife, Brazil; Department of Veterinary Medicine, University of Bari, 70010 Valenzano, Italy; Hospital Veterinario de Virreyes and Enfermedades Parasitarias, Universidad de Buenos Aires, Buenos Aires, Argentina; Programa Institucional Biodiversidade e Saúde, Fundação Oswaldo Cruz, 21040360 Rio de Janeiro, Brazil; Department of Parasitology, Faculty of Veterinary Medicine, Kasetsart University, 10903 Bangkok, Thailand; Department Veterinary Parasitology, Bombay Veterinary College, Maharashtra Animal and Fishery Sciences University, Parel, Mumbai, 400012 Maharashtra India; Faculty of Veterinary Medicine, Vietnam National University of Agriculture, Hanoi, Vietnam; Department of Veterinary Tropical Diseases, Faculty of Veterinary Science, University of Pretoria, Pretoria, Onderstepoort South Africa; Department of Companion Animal Medicine and Surgery, Faculty of Veterinary Medicine, Universiti Putra Malaysia, 43400 UPM Serdang, Malaysia; Bayer Animal Health GmbH, 51368 Leverkusen, Germany

**Keywords:** Dogs, Cats, Parasites, Tropics, Anthelmintic, Deworming, Recommendations, Council, Heartworm

## Abstract

This letter advises the imminent formation of the Companion Animal Parasites Council for the Tropics (CAPCT). The CAPCT consists of region-specific (e.g., Asia-Pacific, Latin America and Caribbean, Africa) experts comprising academics, veterinarians, parasitologists, physicians and allied industry partners that will work together to inform, guide and develop best-practice recommendations for the optimal diagnosis, treatment and control of companion animal parasites in the tropics, with the aim of protecting the health of pets and that of the public.

## Letter to the editor

### The role of companion animal parasite councils

Both the European Scientific Council of Companion Animal Parasites (ESCCAP) and the Companion Animal Parasite Council (CAPC) in the United States are independent, non-profit making organisations consisting of experts in the field of parasitology and public health from across Europe and the United States, respectively. Initially convened in 2002 (CAPC) and 2005 (ESCCAP), the role of these organisations is to develop recommendations for the optimal treatment and control of companion animal parasites with the aim of protecting the health of pets and the health of the public by reducing the risk of zoonotic parasite transmission. The websites comprehensively present the life cycles, diseases, diagnosis, control, prevalence and distribution maps of a wide variety of endoparasites and ectoparasites of dogs and cats. In so doing, the guidelines aim to increase awareness and educate practicing veterinarians and public health professionals of the most relevant parasites within each region and provide guidance for specific best-practice procedures to protect pets and the public from parasitic infections. For the majority of globally distributed parasites that the recommendations for the geographic regions CAPC and ESCCAP represent, there is overlap, owing to commonalities in mode of transmission and risks of exposure of the respective parasites to pets and humans. For others that are restricted geographically, for example the trematode *Nanophyetus salmincola* in the Pacific Northwest of the United States and Canada and *Heterobilharzia americana*, the cause of canine schistosomiasis in south-eastern United States, information and guidelines are highly region-specific. Whenever possible to do so, the guidelines put forward by experts of CAPC and ESCCAP are evidence-based, extracted from comprehensive well-resourced epidemiological and clinical-based studies and updated frequently to cover the latest research.

### The “tropics” are unique

A plethora of arguments stand in favour of developing companion animal parasite prevention and best-practice guidelines for the regions spanning Southeast Asia, the Pacific Islands, Northern Australia, South Asia (including India), Latin America and Africa, otherwise known as the ‘tropics’, the geographical area between the Equator and the Tropics of Cancer and Capricorn. The region is predominantly characterised by hot, humid weather, but also includes other climatic zones, namely the seasonally dry tropics, mountainous and less commonly, arid zones. These climatic conditions support a diverse range of pathogens and vectors of medical and veterinary importance, whose transmission and geographical distribution are closely linked to regional temperature, rainfall, humidity and soil type. The wet tropics in particular, favour an unrivalled animal and plant biodiversity. The high burden of disease transmission is exacerbated by the fact that despite varied economic, political and social histories, almost all of the tropical countries remain underdeveloped with the majority being classified as low to middle income countries by The World Bank (http://data.worldbank.org/about/country-and-lending-groups). Transmission of parasites is therefore particularly high in these less-industrialized nations. In addition, the demographics of dog and cat populations in these respective regions and countries are far from being comparable to Europe or North America. The co-existence of true companion animals with semi-domesticated and stray animals alike, contribute to the challenge of parasite control programmes. Veterinary awareness and treatment of animals may be lacking and environmental and sanitary conditions conducive for their transmission [1, 2]. Many canine and feline parasites are also important zoonoses including species of *Toxocara*, *Ancylostoma* and *Echinococcus.* The diseases caused by these parasites are recognised by the WHO/FAO/OIE as ‘neglected zoonotic diseases’ that form under the umbrella of ‘neglected tropical diseases’ (WHO, http://www.who.int/neglected_diseases/en). Their strategic control requires the application of integrated, multidisciplinary input involving both veterinarians as well as public health practitioners. The term “neglected” highlights diseases that affect mainly poor and marginalised populations in low-resource settings that receive little attention in terms of investment in research towards their surveillance, treatment and control [3].

For example, the higher infection pressure of parasites within the tropics, as distinct from cooler climes, can be demonstrated using *Toxocara* spp. (a globally prevalent parasite). Toxocariasis is an important zoonosis that may manifest as visceral larva migrans, ocular larva migrans, or as non-specific, mild symptoms referred to as ‘covert’ or ‘common’ toxocariasis. Toxocariasis has been shown through seroprevalence studies to be especially prevalent among children from socio-economically disadvantaged populations [4], especially those within the tropics [5]. Seroprevalence of toxocariasis among children has been reported at 33 % in Kashmir, India [6]; 49 % in the Philippines [7]; between 15.5 and 17.8 % in Brazil [8, 9] and as high as 47.5 % among populations in Colombia [10]. This is in contrast to seroprevalence rates of 2.4 % in Denmark [11], 6.6 % in Austria [12], 0–4.2 % in children in Spain [13] and 8.6 to 13.9 % in the United States [14]. *Toxocara* eggs are capable of surviving for several years in optimal environments [15] and therefore high concentrations of infective stages are likely to build up in any area frequented by patent-parasitized dogs. Uncontrolled and untreated dog and cat populations coupled with poor sanitation can lead to heavily contaminated environments. Warm climatic conditions provide ideal transmission opportunities, not only to the occasionally-walked apartment-dwelling dog, but more importantly to the child that shares the animals’ environment.

### Region-specific parasites begets region-specific knowledge

For other parasites, that are more restricted in distribution, knowledge about their impact as an agent of veterinary or human disease relies largely on local research and knowledge. The prevalence of these parasites may depend solely on the distribution of region-specific intermediate hosts (vectors) or be strongly influenced by sylvatic factors or anthroponotic behaviours and therefore necessitate more specific guidelines for their diagnosis and control. For example, in areas where *Dioctophyme renale*, the giant kidney worm of dogs is endemic (including South America, China and parts of Southeast Asia), patients may not be appropriately diagnosed or treated if veterinarians fail to consider the parasite as a differential cause of chronic dysuria and haematuria and occasionally, peritonitis. In Southeast Asia, control of *Clonorchis sinensis* and *Opsithorchis viverrine* relies on educating owners not to provide their animal access to raw fish. For others, differences in pre-patent period may also have important implications on timing of anthelmintic administration. *Ancylostoma ceylanicum* for example, has emerged as the dominant species of hookworm infecting dogs and cats in the Asia Pacific region with a reported prevalence of between 35 and 72 % in India [16, 17], 92 % in Thailand [18], 52 % in Malaysia [19], 69–77 % in Laos [20, 21], and over 90 % in dogs in Cambodia [22]. In addition to causing anaemia in dogs, this hookworm species is recognised as an important emerging zoonosis [22]. A specific feature of the biology of *A. ceylanicum* in dogs and cats is its relatively short pre-patent period of only 14 days compared with other major helminths [23]. In heavily contaminated environments, anthelmintics with pure adulticidal activity administered at frequencies of any greater than once every three months, as currently recommended on most such products in Asia, will ultimately fail to have a significant effect on environmental hookworm burdens, the frequency of re-infection and therefore the level of morbidity in the animal (as well as in humans).

The importance of distinguishing *Dirofilaria immitis* from the relatively benign subcutaneous nodule worm, *Dirofilaria repens* in areas where these filarial worms are co-endemic is critical for companion animal veterinarians. For example, in Vietnam, northern India and Thailand, veterinarians must be vigilant in their choice of antigen test kit for detecting heartworm infection and with correctly identifying microfilaria in blood films; and combining these with the individual’s clinical presentation and ancillary test results, prior to making a definitive diagnosis of heartworm disease. Furthermore, in many of these heartworm-endemic countries (e.g., Malaysia, Brazil and Vietnam), the compound melarsomine (registered as an adulticide), is either unavailable or rarely utilised as part of the heartworm treatment regime. Instead, ‘slow kill’, using macrocyclic lactones only, a method strongly discouraged by the American Heartworm Society (https://heartwormsociety.org), is routinely practiced throughout the tropics. “Slow kill” is not only ineffective, but leads to the selection of macrocyclic lactone resistant sub-populations of heartworms [24].

### Changing trend of companion animal ownership

Companion animal populations can be divided into two major categories - pets (owned and supervised) and strays. Strays may be further classified according to their dependence on humans, namely unowned free-roaming (abandoned and feral), and owned free-roaming (community dogs) [25]. In the United States and the majority of Europe, legislation promoting responsible pet ownership assures that the majority of companion animals are indeed ‘pets’. In Europe and the United States there is one dog or cat to every 3 to 4 households (https://www.avma.org, 2012; http://www.fediaf.org, 2010). In contrast, three quarters of the dog population in developing regions of the tropics may be classified as stray or free-roaming community dogs. These may equate to as high as 1 dog to 5 people in Kathmandu, Nepal and Bali, Indonesia; 1 dog to 4 people in the Philippines and Bolivia and 1 dog to 3 people in rural Mexico [26]. These animals contaminate the environment with excreta that are a source of parasite eggs and larvae capable of infecting pet dogs and humans. In recent years however, dog ownership rates in these now developing economies are on the rise, as are rapid changes in the nature of companion animal ownership [27]. For example, India has one of the world’s lowest rates of dog ownership: 4 dogs per 1,000 people, however, in the last 5 years, dog ownership rates have more than doubled (+58 %) with an estimated pet dog population of 10 million, the fastest growth rate of the 53 countries surveyed (http://www.euromonitor.com/pet-care, 2014). Similar trends were recorded for the Philippines (+38 %), Venezuela (+30 %), Argentina (+20 %) and Brazil (+14.3 %). In these regions, the rapidly urbanising middle class appear to be working more, earning more and living in city apartments sharing close relationships with pet dogs. The close human-animal bond has undoubted mental and physical benefits [2], however, these come at a potential cost of acquiring parasitic zoonoses.

### The role and knowledge of veterinarians on the importance of parasite prevention

The benefits of economic growth and affluence also place an increasingly higher expectation and demand on veterinary practitioners by animal owners for improved standards of service in healthcare for their pets, and protection of their family from acquiring zoonoses. In developing nations, veterinarians are deemed at the forefront when managing the risk of zoonotic diseases [28]. The profession has an ethical and legal professional duty of care to diagnose and prevent zoonotic diseases in animals as well as to refer a pet owner to a physician if potential risk of zoonotic disease transmission exists [29], and this responsibility extends to the provision of advice about parasite prevention.

Recently, region-specific guidelines for immunization of dogs and cats were published by the World Small Animal Veterinary Association Vaccination Guideline Group [30]. The report highlighted unique challenges faced by companion animal practitioners in Asia, which included insufficient resources for research, training and diagnosis into small animal infectious diseases and limited awareness of veterinarians on global trends on small animal vaccinology. Practicing veterinarians in the tropics are likely to face similar challenges with regard to their ability to accurately diagnose, treat and control companion animal parasites. For example, in the United States, Europe and Australia, veterinarians follow guidelines outlined by CAPC and ESCCAP, for the control of hookworm and *Toxocara* spp. in puppies, which consists of de-worming with appropriate anthelmintics at 2 weekly intervals until 8 weeks of age, and monthly thereafter. These recommended ‘intervals’ vary according to country. For instance, in Vietnam, Philippines and Malaysia veterinarians often de-worm puppies at 4, 8 and 12 weeks of age, followed by 2 to 4 times annually. Recommended de-worming intervals also vary according to local manufacturers’ guidelines, the perceived ‘risk’ of parasite infection by the veterinarian, their knowledge of the parasite’s life cycle and its clinical and zoonotic significance. In much of the tropics, resources to fund studies to address these issues are limited. Nevertheless, the past decade has seen a moderate increase in the published literature of research associated with companion animal parasites in tropical countries. Much of this has been focused on the surveillance and control of diseases that are zoonotic (e.g., echinococcosis, rabies, toxocariasis), rather than aiming to improve health standards of the animals themselves. There is, therefore, a significant gap in our knowledge about the significance and distribution of clinically important parasitic diseases of dogs and cats in much of the tropics. Furthermore, the average veterinarian’s ability to access relevant and up-to-date information about these diseases for their region is also a major factor that limits their ability to translate the research into everyday practice.

The combination of warmer climate, enhanced parasite life cycles, poor environmental sanitation and higher environmental contamination rates lead to significantly increased infection pressure for all animals and potentially pose a serious risk to the public. This risk extends to pets that are inadequately treated as puppies and adults, even if ‘occasionally’ walked outdoors. It is the opinion of the authors that there needs to be questions raised about the appropriateness of ‘standard’ parasite prevention protocols currently advocated by most companion animal clinicians in the tropics and a paradigm shift in the thinking of veterinarians with regard to treating dogs and cats.

### Towards a solution

We propose formation of the Companion Animal Parasites Council for the Tropics (CAPCT). The CAPCT will consist of regional (e.g. Asia-Pacific, Latin America and Caribbean, Africa) experts comprising academic veterinarians, parasitologists, physicians and allied industry partners that will work together to inform, guide and make best-practice recommendations for the diagnosis, treatment and control of companion animal parasites in the tropics, with the aim of protecting animal and human health. The organization will seek input through close collaboration with the practicing veterinarians, medical and public health professions, regional government and non-for-profit bodies. In addition to biannual meetings, the Council will organize veterinary and public outreach events in each region to facilitate transfer of knowledge through local lectures, workshops, symposia within local conferences and webinars to primary stakeholders such as veterinarians and breeders. General and region-specific recommendations for the treatment and control of companion animal parasites will be developed and made available through a dedicated, freely accessible website.

## Abbreviations

CAPCT: Companion Animal parasites Council for the Tropics
CAPC: Companion Animal Parasite Council
ESCCAP: European Scientific Council of Companion Animal Parasites
OIE: World Organisation for Animal Health
FAO: Food Agricultural Organisation
WHO: World Health Organisation
